# Design, Synthesis, and Characterization of Novel Bis-Uracil Chitosan Hydrogels Modified with Zinc Oxide Nanoparticles for Boosting Their Antimicrobial Activity

**DOI:** 10.3390/polym15040980

**Published:** 2023-02-16

**Authors:** Rana A. Alharbi, Fahad M. Alminderej, Nouf F. Al-Harby, Noura Y. Elmehbad, Nadia A. Mohamed

**Affiliations:** 1 Department of Chemistry, College of Science, Qassim University, Buraidah 51452, Saudi Arabia; 2 Department of Chemistry, Faculty of Science and Arts, Najran University, Najran 55461, Saudi Arabia; 3 Department of Chemistry, Faculty of Science, Cairo University, Giza 12613, Egypt

**Keywords:** chitosan, crosslinking, ZnO nanoparticles, antimicrobial activity, cytotoxicity

## Abstract

A new series of hydrogels was successfully prepared by incorporating various substituted bisuracil (R-BU) linkages between chitosan Schiff’s base chains (R-BU-CsSB) and between chitosan chains (R-BU-Cs). After protection of the amino groups of chitosan by benzaldehyde, yielding chitosan Schiff’s base (CsSB), the reaction with epichlorohydrin was confined on the -OH on C6 to produce epoxy chitosan Schiff’s base (ECsSB), which was reacted with R-BU to form R-BU-CsSB hydrogels, and finally, the bioactive amino groups of chitosan were restored to obtain R-BU-Cs hydrogels. Further, some R-BU-Cs-based ZnO nanoparticle (R-BU-Cs/ZnONPs) composites were also prepared. Appropriate techniques such as elemental analysis, FTIR, XRD, SEM, and EDX were used to verify their structures. Their inhibition potency against all the tested microbes were arranged as: ZnONPs bio-composites > R-BU-Cs hydrogels > R-BU-CsSB hydrogels > Cs. Their inhibition performance against Gram-positive bacteria was better than Gram-negative ones. Their minimum inhibitory concentration (MIC) values decreased as a function of the negative resonance effect of the substituents in the aryl ring of R-BU linkages in the hydrogels. Compared with *Vancomycin*, the ZnONPs bio-composites showed superior inhibitory effects against most of the tested Gram-negative bacteria, all inspected Gram-positive ones, and all investigated fungi.

## 1. Introduction

Antibiotics play a significant role in preventing and controlling the spread of pathogenic microorganisms. Nevertheless, infections continue to be a major cause of diseases and mortality in contemporary societies due to the emergence of antibiotic resistance, new pathogenic strains, and a lack of effective therapeutics [1]. The utilization of novel substances as options for therapy and disinfection, such as antimicrobial polymers, has gained popularity [2].

Chitosan (Cs), as one of the most important natural biopolymers, has attracted a great attention due to its unique features such as renewability, bio-degradability, bio-compatibility, non-toxicity, hydrophilicity, high viscosity, film-forming ability, polyelectrolyte behavior, antioxidant properties, antimicrobial activity, and fat, mineral, and dyes adsorption properties [3,4,5,6,7].

The disadvantages of Cs include its high solubility in acidic media, low heat resistance, and restricted porosity, resulting in a significant obstacle for many of its uses. These drawbacks could be addressed utilizing several chemical modification processes: first, by grafting copolymerization using a functional monomer as side chains, including N-hydroxy ethyl acrylamide [8], 2-(acryloyloxy) ethyl trimethylammonium chloride [9] and acrylonitrile [10]. Second, by blending with a synthetic polymer including poly(vinyl alcohol) [11], polyacrylic acid [12], and polyethylene glycol [13]. Third, by cross-linking through the creation of bridges between its chains followed by the formation of a network using various cross linkers such as terephthaloyl diisothiocyanate [14] and aminobenzhydrazide [15]. Finally, by substitution reaction using a functional moiety including amino salicylic acid [4] and benzhydrazide [16]. Further, all these modification processes improved the antimicrobial activity of chitosan [4,8,9,10,11,12,13,14,15,16].

Uracil is a naturally occurring component, a member of the pyrimidine family [17], and one of the four nucleobases of the biopolymer RNA [17,18,19,20]. Thus, it is one of the most significant structures in life [21], and it has demonstrated a class of molecules that continues to attract the attention of the medicinal chemists, organic chemists, and photobiologists. Three bis-uracil derivatives were prepared via reacting 6-[(dimethylamino)methyleneamino]-1,3-dimethyluracil with three different aldehydes: p-methoxybenzaldehyde, p-nitrobenzaldehyde, and 2-thiophenecarboxaldehyde. These derivatives showed from moderate to good antibacterial properties (minimum inhibition concentration (MIC) ranged from 25 to 100 mg/mL) against *Klebsiella pneumoniae, Staphylococcus aureus*, and *Pseudomonas aeruginosa* [22]. Modified chitosan samples with various pendant (E)-5-((4-acetylphenyl)diazenyl)-6-aminouracil (APAU) groups’ (APAU) content were prepared by forming Schiff base linkages between chitosan and the pendant groups. These samples were active against *S. aureus*, with an MIC of 390 mg/mL, half that of virgin chitosan at 780 mg/mL. Further, they exhibited MIC values of 23.4 mg/mL against *K. pneumoniae* and *E. coli*, much better than that of plain chitosan (187.5 mg/mL) and close to that of commercial *Fluconazole* (11.7 mg/mL). A strong synergetic antimicrobial effect was observed in chitosan with a higher APAU content [23].

In our previous work, a uracil-modified chitosan based adsorbent has shown a high adsorption efficiency for the removal of Congo red dye from its aqueous solution [24].

Notably, metal oxide nanoparticles (MONPs) are among the most investigated nanomaterials in the fight against multidrug-resistant bacteria [25,26]. In comparison to other MONPs, zinc oxide nanoparticles (ZnONPs), as a type of low-cost and low-toxicity nanomaterial, have outstanding biomedical applications such as drug delivery, antibacterial, diabetes treatment, anti-inflammation, wound healing, and bioimaging [27]. 

The Cs/ZnO nanorods demonstrated an antifouling efficiency of 75–90% against the growth of algae [28]. Cs/ZnO nanocomposites showed a good antifungal activity against pathogenic *Candida albicans* [29]. The Cs-encapsulated ZnO hybrid composite exhibited outstanding antibacterial action against *Escherichia coli* [30].

In the present work, we have prepared a series of novel bisuracil crosslinked chitosan hydrogels. Some bio-composites impregnated with ZnONPs in various weight ratios have been also prepared. The structure of the hydrogels and ZnONPs bio-composites was characterized using elemental analyses, FTIR, XRD, EDS, and SEM. The hydrogels and ZnONPs bio-composites’ efficacy against different types of fungi, Gram-positive, and Gram-negative bacteria was examined. Their cytotoxicity was evaluated as a part to expand their applications.

## 2. Materials and Methods

### 2.1. Materials

Cs (1.0–3.0 × 10^5^ g mol^−1^ molecular weight and 98% degree of deacetylation) was made from the shell of shrimps and crabs Pandalus borealis and purchased from Acros Organics (USA). ZnONPs (product No. NCZ4701, powder, and 20 nm particle size) were supplied by nanochemazone (Canada). Epichlorohydrin was obtained from PanReac. AppliChem-ITW Reagent (USA). 6-Amino-1,3-dimethyluracil, benzaldehyde, 2-chlorobenzaldehyde, 4-chlorobenzaldehyde, 2-methoxybenzaldehyde, formaldehyde, and 2-nitrobenzaldehyde were purchased from Sigma-Aldrich (Germany). XTT (2, 3-bis [2-methyloxy-4-nitro-5-sulfophenyl]-2H-tetrazolium-5-carboxanilide) was obtained from Sigma (St. Louis, MO, USA).

### 2.2. Methods

#### 2.2.1. Synthesis of Substituted Bisuracil (R-BU) Derivatives

**Scheme 1:**
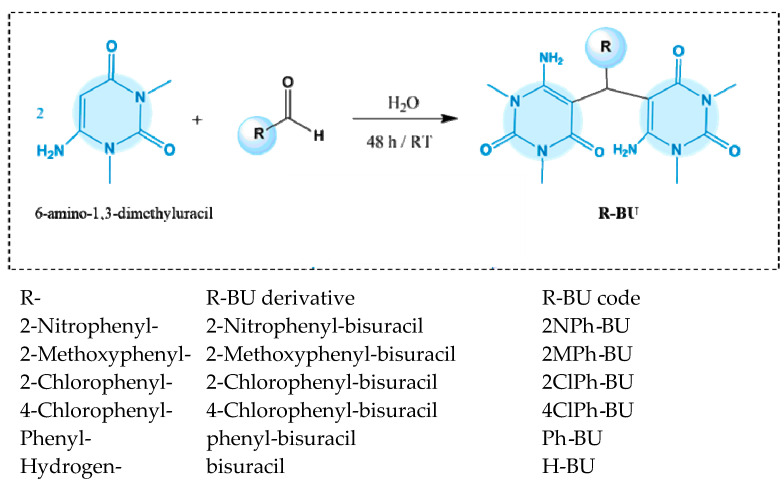
Preparation of R-BU derivatives.

A series of R-BU derivatives has been prepared according to the method descried previously [20], by stirring 6-amino-1,3-dimethyluracil with various substituted aldehydes (R-CHO) in water, as a reaction medium, at room temperature for 48 h as illustrated in Scheme 1. Th elemental analysis and the characteristic FTIR peaks of the produced R-BU derivatives were in good agreement with those reported previously [20].

#### 2.2.2. Synthesis of Novel R-BU-CsSB and R-BU-Cs Hydrogels

A series of novel R-BU-CsSB and R-BU-Cs hydrogels has been prepared via an essential similar four-step procedure as follows:

Step 1: Benzaldehyde (20 mL) was added slowly to the Cs suspension (5 g swollen in 60 mL of MeOH for an hour) at room temperature and stirred for 24 h before being filtered. The formed yield (chitosan Schiff’s base, CsSB) was dried, after being washed several times with MeOH, for 8 h in an oven at 55 °C. This step was performed to protect the amino groups of chitosan and confine the modification reaction on the primary -OH group at C6 in chitosan [31].

Step 2: Epichlorohydrin (10 mL) was slowly added to 4 g of CsSB that was stirred in 120 mL of aqueous NaOH solution (0.001 mol L^−1^) at room temperature for 15 min in order to swell and alkalize. The stirring was continued for an additional 6 h, and the resulting epoxy chitosan Schiff’s base (ECsSB) was collected by filtration, rinsing it many times with water then MeOH and drying it at 55 °C [32].

Step 3: R-BU (1 mmol) was dissolved in 25 mL of EtOH and then mixed with ECsSB suspension (2.35 g, 2 mmol), which was swollen in 60 mL aqueous NaOH solution (0.001 mol L^−1^), and the resulting mixture was stirred at room temperature overnight. The yield, R-BU-chitosan Schiff’s base (R-BU-CsSB), was obtained through filtration, repeated washing with H_2_O and then MeOH, and finally drying at 55 °C.

**Scheme 2:**
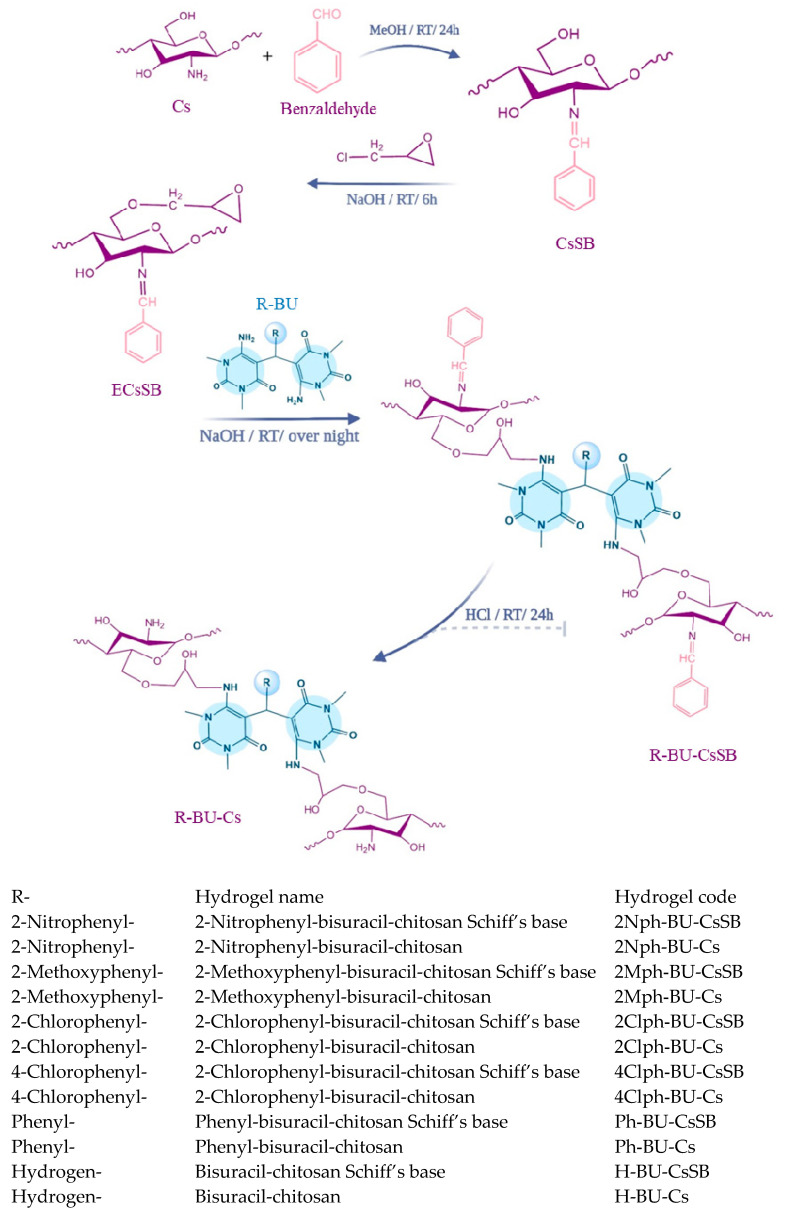
Synthesis of R-BU-CsSB and R-BU-Cs hydrogels.

Step 4: R-BU-CsSB (2 g) was stirred in ethanolic HCl solution (60 mL, 0.24 mol L^−1^) at room temperature for 24 h. Then, aqueous sodium carbonate solution (1 wt%) was used to neutralize the reaction mixture until pH 7 was reached. The resulting R-BU-chitosan (R-BU-Cs) was filtered, rinsed with water then ethanol, and dried to a constant weight at 55 °C (Scheme 2).

#### 2.2.3. Synthesis of 2Nph-BU-Cs/ZnONPs and 2Mph-BU-Cs/ZnONPs Composites

**Scheme 3:**
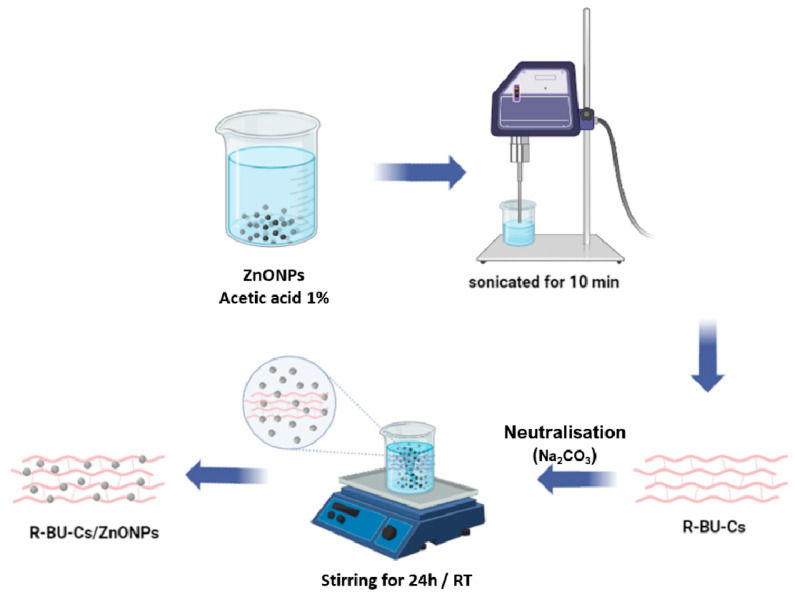
Synthesis of 2Nph-BU-Cs/ZnONPs and 2Mph-BU-Cs/ZnONPs composites.

Firstly, three different precalculated quantities of ZnONPs were dissolved separately in aqueous acetic acid solution (1%) via sonication for 10 min. Then, the formed solutions were added to a fixed weight of 2Nph-BU-Cs and 2Mph-BU-Cs hydrogels (0.25 g) that swelled in 30 mL of distilled water and stirred for 24 h at room temperature (Scheme 3). Afterwards, the acidic media were neutralized using Na_2_CO_3_ solution. The ZnONPs bio-composites were filtered, washed with H_2_O followed by EtOH, and dried at 55 °C. The used ZnONPs concentrations were 1, 3, and 5% based on the weight of the hydrogels. Thus, six composites, designated as 2Nph-BU-Cs/ZnONPs-1%, 2Nph-BU-Cs/ZnONPs-3%, 2Nph-BU-Cs/ZnONPs-5%, 2Mph-BU-Cs/ZnONPs-1%, 2Mph-BU-Cs/ZnONPs-3%, and 2Mph-BU-Cs/ZnONPs-5%, were produced.

### 2.3. Measurements

#### 2.3.1. Elemental Analysis

C, H, N, S Analyzer (Perkin Elmer, Model 2410 series II, Waltham, MA, USA) were utilized for determining the elements of the dried Cs, CsSB, ECsSB, R-BU-CsSB, and R-BU-Cs hydrogels.

#### 2.3.2. FTIR Spectroscopy

FTIR spectroscopy measurements of the dried Cs, CsSB, ECsSB, R-BU-CsSB, and R-BU-Cs hydrogels as well as 2Nph-BU-Cs/ZnONPs and 2Mph-BU-Cs/ZnONPs composites were recorded on Agilent Technologies FTIR Spectrometer (Cary 600 Series, Santa Clara, CA, USA) in the wavenumber range from 4000 to 400 cm^−1^.

#### 2.3.3. X-ray Diffractometry

X-ray diffractometer (Joel JDX-8030, Akishima, Japan) was utilized to investigate the morphology of the dried Cs, CsSB, ECsSB, R-BU-CsSB, and R-BU-Cs hydrogels as well as 2Nph-BU-Cs/ZnONPs and 2Mph-BU-Cs/ZnONPs composites at a diffraction angle (2θ) throughout a range of 5 to 90°, with a 5° min^−1^ speed.

#### 2.3.4. Scanning Electron Microscopy

A scanning electron microscope (Joel JSEM-6010PLUS/LV, Akishima, Japan) was employed to image the surface topography of the dried Cs, CsSB, ECsSB, R-BU-CsSB, and R-BU-Cs hydrogels after they have been coated with gold, at a magnification of 7000× and at a 15 kV accelerating voltage.

#### 2.3.5. EDS Measurements

The ZnONPs that were dispersed inside the matrix of the dried 2Nph-BU-Cs hydrogel (2Nph-BU-Cs/ZnONPs-5%) were detected using (Joel JSEM-6010PLUS/LV, Akishima, Japan) equipped with EDS.

#### 2.3.6. Determination of the Minimal Inhibitory Concentration (MIC) Using XTT Assay

MIC is the lowest concentration of the investigated samples and the standard antibiotics (*Vancomycin and Amphotericin B*) that totally inhibits the microbial growth. MIC values were determined using the micro-dilution technique [33]. A microtiter plate reader (Tecan Sunrise absorbance reader; Tecan UK, Reading, United Kingdom) was used to quantify the colorimetric change in the XTT reduction test.

#### 2.3.7. Cytotoxicity Evaluation Using Viability Assay

The cytotoxicity of the prepared samples was measured against a normal human lung fibroblast cells line (MRC-5 cells) using the viability assay that was previously reported [34]. The cellular morphology was observed using an inverted microscope (CKX41; Olympus, Tokyo, Japan) equipped with the digital microscopy camera to capture the images representing the morphological changes compared to the control cells. The cytopathic effects (morphological alterations) were microscopically observed at 100×.

## 3. Results and Discussion

### 3.1. Synthesis of R-BU-CsSB and R-BU-Cs Hydrogels and ZnONPs Bio-Composites

R-BU-CsSB and R-BU-Cs hydrogels (Scheme 2) were obtained at the final stage of a reaction consisted of four consecutive steps. In the first step, the primary amine groups in Cs were protected by condensing them with the C=O groups of the benzaldehyde, producing CsSB. Hence, in the second step, the reaction with epichlorohydrin was confined on the primary hydroxyl groups at C6, creating ECsSB. Afterwards, epoxy rings of ECsSB, in the third step, were facilely opened using nitrogen-rich R-BU derivatives via their lone pair of electrons, yielding R-BU-CsSB hydrogels. At the end, in the fourth step, the amino groups of Cs were recovered by the removal of the protection in an acidic medium, producing R-BU-Cs hydrogels. Thus, the combination of a nitrogen-rich R-BU, as linkages between the CsSB chains (R-BU-CsSB hydrogels) and between Cs chains (R-BU-Cs hydrogels), hydroxyl groups, and the regained amino groups at Cs, will greatly enhance the cationic sites that possess a high capacity to inhibit the microbial growth. Further, some ZnO nanocomposites based on 2Nph-BU-Cs and 2Mph-BU-CS were made using three different concentrations of ZnONPs of 1, 3, and 5% (based on the weight of the hydrogel), producing 2Nph-BU-Cs/ZnONPs-1%, 2Nph-BU-Cs/ZnONPs-3%, 2Nph-BU-Cs/ZnONPs-5%, 2Mph-BU-Cs/ZnONPs-1%, 2Mph-BU-Cs/ZnONPs-3%, and 2Mph-BU-Cs/ZnONPs-5%.

### 3.2. Characterization of R-BU-CsSB and R-BU-Cs Hydrogels and ZnONPs Bio-Composites

#### 3.2.1. Elemental Analysis

The elemental analysis values of all R-BU-CsSB and R-BU-Cs hydrogels were recorded in Table 1. In regard to %C and %N, it could be noted that the %C value of CsSB (62.80) increased at the expense of its %N value (5.70) in comparison to those of the virgin Cs (%C, 44.90 and %N, 8.61). This confirms the successful amalgamation of the carbon- rich benzaldehyde moieties into the repeating units of Cs. On contrast, the %N values of the 2Nph-BU-CsSB, 2Clph-BU-CsSB, 4Clph-BU-CsSB, ph-BU-CsSB, H-BU-CsSB, and 2Mph-BU-CsSB were 13.14, 11.73, 11.84, 12.10, 13.35, and 11.68, respectively, which were higher than those obtained for both CsSB (5.70) and ECsSB (4.48). This is due to various nitrogen-rich R-BU moieties that were incorporated into the repeating units of ECsSB. This was additionally proved by the presence of chlorine element in the elemental analysis of 2Clph-BU-CsSB (3.42%) and 4Clph-BU-CsSB (3.81%). Moreover, a decrease in the %C values accompanied with further increases in the %N values of the 2Nph-BU-Cs (C, 50.56 and N, 14.47), 2Clph-BU-Cs (C, 51.14 and N, 13.00), 4Clph-BU-Cs (C, 51.09 and N, 12.98), ph-BU-Cs (C, 53.45, and N, 13.46) H-BU-Cs (C, 49.09 and N, 14.85), and 2Mph-BU-Cs (C, 53.11 and N, 12.92) in comparison to 2Nph-BU-CsSB (C, 54.61 and N, 13.14), 2Clph-BU-CsSB (C, 55.17 and N, 11.73), 4Clph-BU-CsSB (C, 55.12 and N, 11.84), ph-BU-CsSB (C, 57.50 and N, 12.10), H-BU-CsSB (C, 53.89 and N, 13.35), and 2Mph-BU-CsSB (C, 56.97 and N, 11.68) were observed. This emphasizes the removing of the benzaldehyde moieties from chitosan part and retrieving its 1^ry^ amino groups.

#### 3.2.2. FTIR Spectroscopy

**Figure 1:**
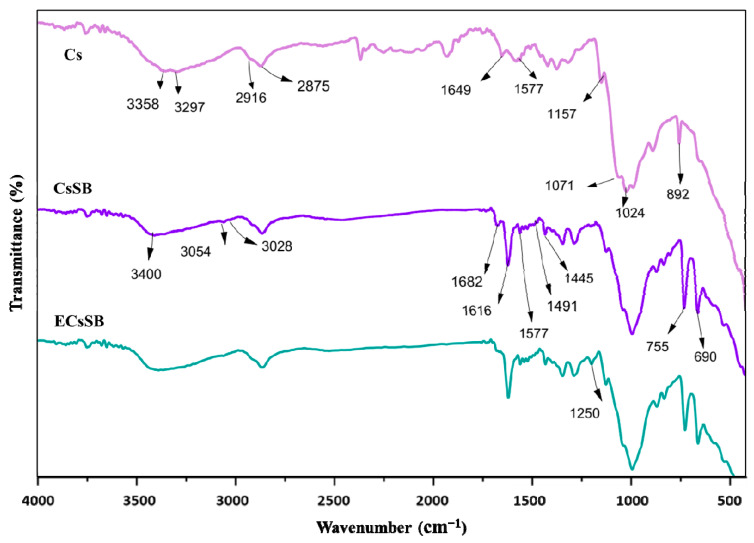
FTIR spectra of Cs, CsSB, and ECsSB.

Figure 1 shows the FTIR spectra of Cs before and after modification. In virgin Cs, between 3700 and 3000 cm^−1^, a very broad absorption peak was observed, corresponding to the stretching vibration of the hydroxyl groups overlapping with that of -NH_2_ groups and their hydrogen bonds. In this wavenumber range, there is a doublet peak at 3358 and 3297 cm^−1^ related to the -NH_2_ groups. The symmetric stretching vibration peaks of the -CH and -CH_2_ groups in the moieties of pyranose appeared at 2916 and 2875 cm^−1^, respectively. Further, two weak peaks appeared at 1649 and 1577 cm^−1^ attributable to amide I and amide II, respectively, confirming the high Cs degree of deacetylation. The four absorption peaks that appeared at 1157, 1071, 1024, and 892 cm^−1^ confirmed the saccharide moieties of Cs [35].

In the CsSB spectrum, the doublet peak corresponding to NH_2_ groups of chitosan disappeared and was replaced by a single one at 3400 cm^−1^, which can be attributed to -OH groups. In addition, some new peaks were observed: at 3054 and 3028 cm^−1^ (C-H, aromatic), at 1682 cm^−1^ (C=N groups), at 1616, 1577, 1491, and 1445 cm^−1^ (C=C, aromatic), and at 755 and 690 cm^−1^ (mono-substituted benzene ring) [24], indicating that all the -NH_2_ groups were consumed during protection with benzaldehyde and confirming the successful formation of CsSB.

In addition to the aforementioned stated peaks for CsSB, the ECsSB spectrum revealed a new peak at 1250 cm^−1^ that is attributed to the moieties of epoxide [16].

**Figure 2:**
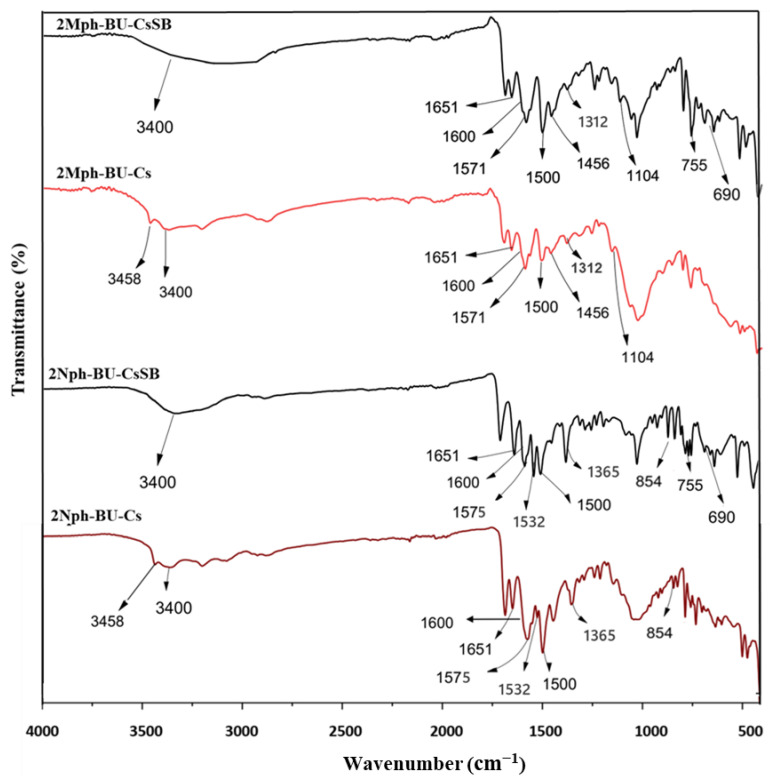
FTIR spectra of 2Mph-BU-CsSB, 2Mph-BU-Cs, 2Nph-BU-CsSB and 2Nph-BU-Cs hydrogels.

In Figure 2, the spectra of 2Nph-BU-CsSB and 2Mph-BU-CsSB revealed the evanescence of the peak at 1250 cm^−1^ that related to the epoxy nuclei. This was accompanied with the apparition of new absorption peaks: at around 1500, 1571–1575, and 1600 cm^−1^ (C=C, aromatic ring) of R-BU moiety for both, at 1532, 1365, and 854 cm^−1^ (-NO_2_ group) for 2Nph-BU-CsSB, and at 1456, 1312, and 1104 cm^−1^ (-OCH_3_ group) for 2Mph-BU-CsSB. The peak appeared around 1651 cm^−1^, corresponding to C=O of BU moiety, overlapping with that of the amide I in chitosan at 1649 cm^−1^. This affirms the completeness of the reaction of ECsSB with 2Nph-BU and 2Mph-BU, respectively. The restoring of the doublet peak of the -NH_2_ groups of chitosan moieties at 3458 and 3400 cm^−1^, in addition to the demise of the of mono-substituted benzene rings absorption bands at 755 and 690 cm^−1^, confirm the elimination of benzaldehyde nuclei to obtain 2Nph-BU-Cs and 2Mph-BU-Cs hydrogels.

**Figure 3:**
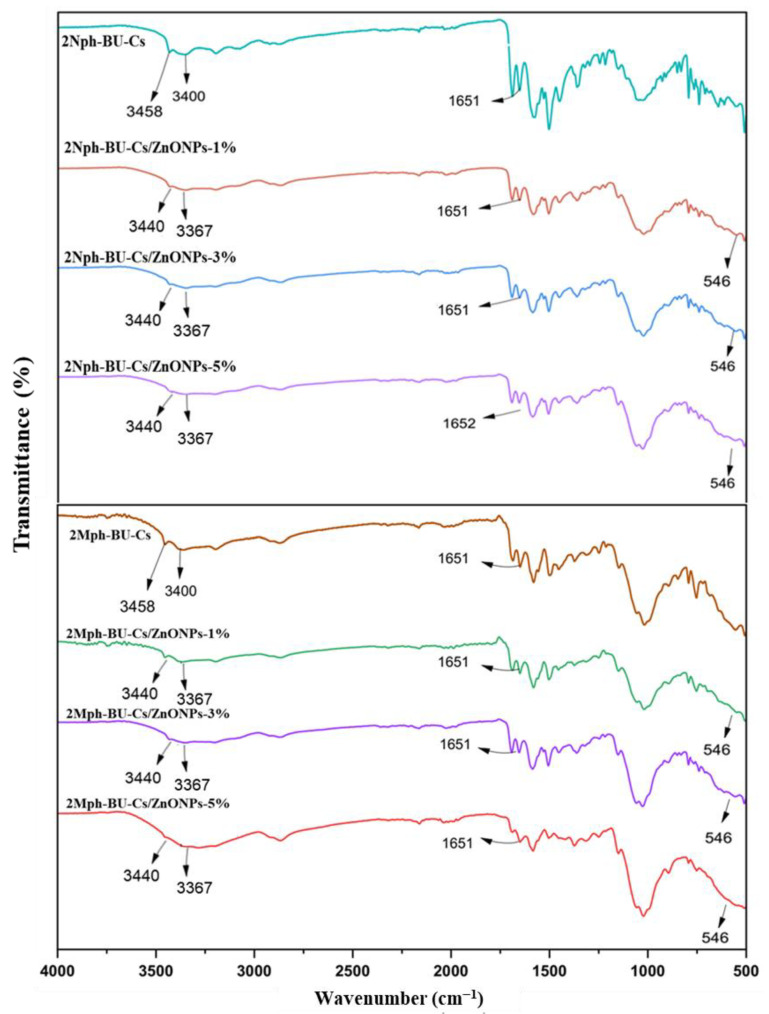
FTIR spectra of 2Nph-BU-Cs, 2Mph-BU-Cs, and their ZnO nanocomposites.

Figure 3 showed the FTIR spectra of 2Nph-BU-Cs/ZnONPs-1%, 2Nph-BU-Cs/ZnONPs-3%, 2Nph-BU-Cs/ZnONPs-5%, 2Mph-BU-Cs/ZnONPs-1%, 2Mph-BU-Cs/ZnONPs-3%, 2Mph-BU-Cs/ZnONPs-5% composites. The doublet peak at 3458 and 3400 cm^−1^ corresponded to NH_2_ bonds in 2Nph-BU-Cs, and 2Mph-BU-Cs appreciably shifted to a lower frequency of 3440 and 3367 cm^−1^, respectively, together with an observable decrease in their intensity in all the ZnONPs bio-composites. Further, the intensity of the peak at 1651 cm^−1^ related to C=O in both 2Nph-BU-Cs and 2Mph-BU-Cs considerably decreased after the incorporation of ZnONPs into their matrices. The spectra also showed a new peak at 546 cm^−1^, attributed to the O-ZnO bond [36]. All these changes prove that ZnONPs interacted with the functional groups of both 2Nph-BU-Cs and 2Mph-BU-Cs.

#### 3.2.3. Powder X-ray Diffractometry (XRD)

**Figure 4:**
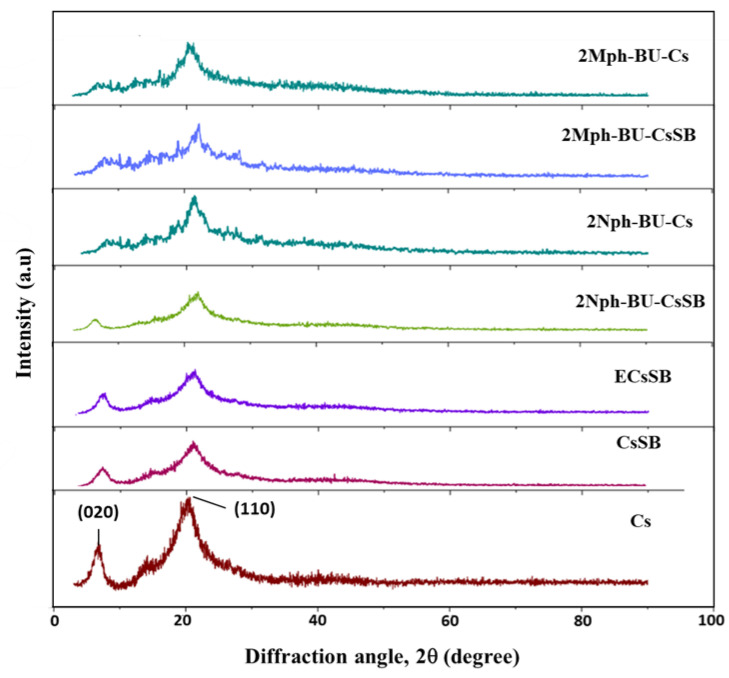
XRD patterns of 2Mph-BU-CsSB, 2Mph-BU-Cs, 2Nph-BU-CsSB, and 2Nph-BU-Cs hydrogels.

XRD was utilized to inspect the changes in the internal structure of chitosan before and after its modification. The XRD patterns of the virgin Cs and representative examples of its hydrogels were illustrated in Figure 4. In Cs, two distinguished peaks close to 2θ = 20° and 10° that were indexed to crystal planes of (110) and (020), respectively [37], were observed attributable to its crystalline nature [35]. This can be attributed to the creation of a lot of hydrogen bonds throughout its chains as a result of the abundance of its hydroxyl and amino groups. In comparison to Cs, the CsSB and ECsSB are less crystalline. This was illustrated not only by lowering the intensity of both these peaks but also by increasing their broadening. Moreover, the near disappearance of the peak at 2θ = 10°, together with a further broadening and reduction in the intensity of the peak at 2θ = 20°, were observed in all patterns of 2Nph-BU-CsSB, 2Nph-BU-Cs, 2Mph-BU-CsSB, and 2Mph-BU-Cs, suggesting their much less crystallinity (Figure 4). The incorporation of the R-BU linkages between the repeating units of CsSB as well as Cs greatly decreased the possibility of the formation of the hydrogen bonds between their chains. This is ascribed not only by the exhausting of their polar groups (-NH_2_ and/or -OH) during modification process but also by separating the Cs chains away from each other.

**Figure 5:**
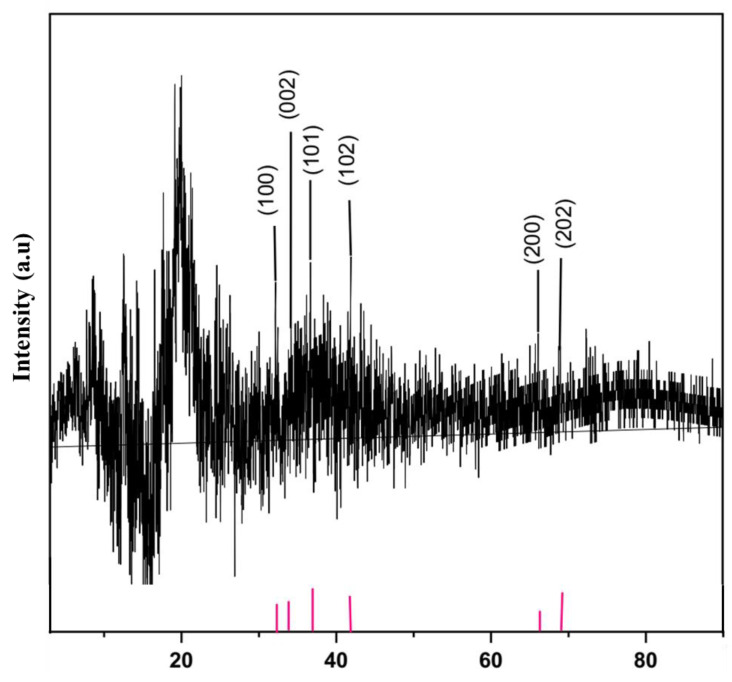
XRD pattern of 2Nph-BU-Cs/ZnONPs-5% composite.

In order to confirm the loading of ZnONPs into the matrices of 2Nph-BU-Cs, XRD measurement was performed for the prepared 2Nph-BU-Cs/ZnONPs-5% composite (Figure 5). Its XRD pattern showed six new peaks, in addition to the amorphous peaks of 2Nph-BU-Cs near 2θ = 20°, at 2θ = 32.2°, 34.76°, 36.37°, 41.8°, 66.14°, and 69.1° that were indexed to crystal planes of (100), (002), (101), (102), (200), and (202), respectively. These peaks and their corresponding crystal planes are in good agreement with those for the pure ZnO as previously reported (JCPDS card no. 36-145) [28,29,38,39,40,41]. This evidences the successful formation of the 2Nph-BU-Cs/ZnONPs composite.

#### 3.2.4. SEM Analysis

**Figure 6:**
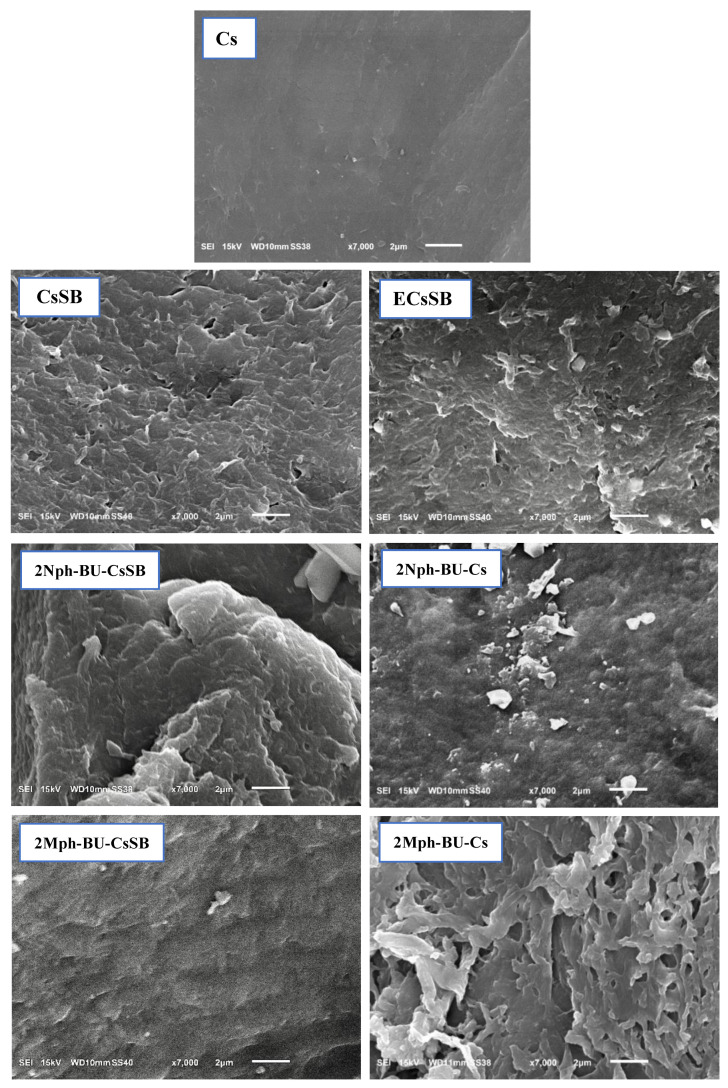
SEM images of 2Mph-BU-CsSB, 2Mph-BU-Cs, 2Nph-BU-CsSB, and 2Nph-BU-Cs hydrogels.

The SEM images of the topographical features of the surfaces of Cs, CsSB, ECsSB, as well as 2Nph-BU-CsSB, 2Nph-BU-Cs, 2Mph-BU-CsSB, and 2Mph-BU-Cs hydrogels are shown in Figure 6. It was observed that the Cs surface was very smooth, while its hydrogels had much more rough surfaces that were composed of lumps of various sizes due to the size differences of the inserted modifier moieties. It can be also noted that the distribution of these lumps in each derivative was homogenous, referring to the fact that the modification of Cs in each step was successfully accomplished.

#### 3.2.5. Energy-Dispersive Spectroscopy (EDS)

**Figure 7:**
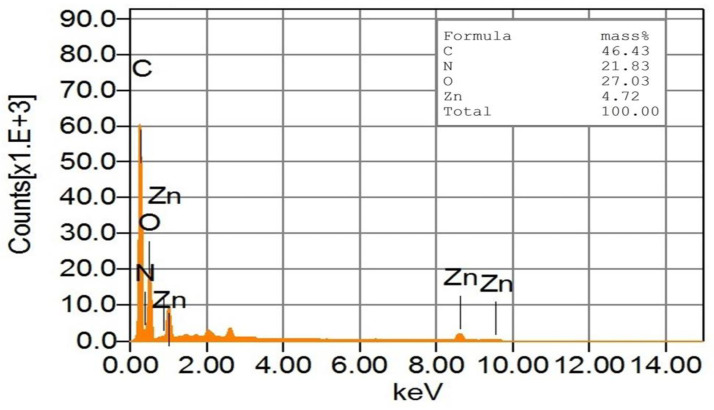
EDS spectrum of the 2Nph-BU-Cs/ZnONPs-5% composite with the percentage of its elements.

**Figure 8:**
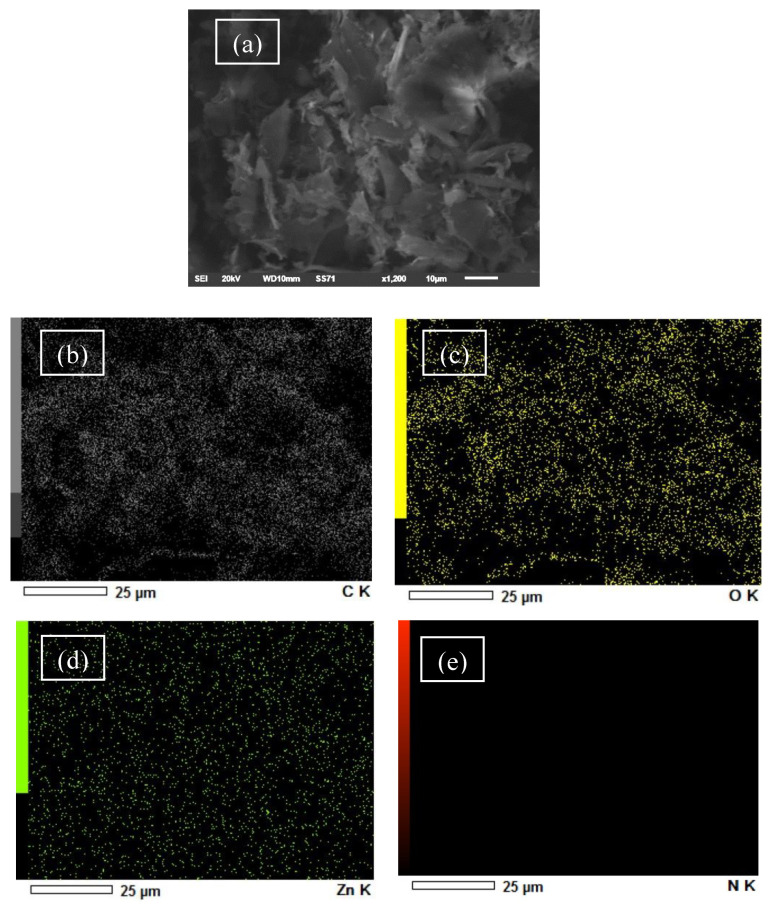
Elemental distribution and mapping analysis of 2Nph-BU-Cs/ZnONPs-5% (**a**) SEM image, (**b**) carbon element, (**c**) oxygen element, (**d**) zinc element, and (**e**) nitrogen element.

The elements in the 2Nph-BU-Cs/ZnONPs-5% composite were identified using EDS. The EDS spectrum (Figure 7) showed the presence of zinc (4.72%), oxygen (27.03%), carbon (46.43%), and nitrogen (21.83%) elements in synthesized nano bio-composite. The detection of these elements indicated the successful formation of the 2Nph-BU-Cs/ZnONPs-5% composite. Figure 8 depicted the distribution of different components in the synthesized nano bio-composite after it was imaged.

### 3.3. Antibacterial Activity

By employing the micro-dilution method, the antibacterial activity of the Cs, CsSB, ECsSB, the prepared hydrogels, and the ZnONPs bio-composites was assessed against some Gram-positive bacteria, namely *Staphylococcus aureus* (*S. aureus* ATCC 25923), *Streptococcus pyogenes* (*S. pyogenes* ATCC 12344)*, Bacillus subtilis* (*B. subtilis* ATCC 6051), and *Staphylococcus epidermidis (S. epidermidis* ATCC 11774), as well as against some Gram-negative bacteria, namely *Escherichia coli (E. coli* ATCC 11775)*, Proteus mirabilis* (*P. mirabilis* ATCC 12453)*, Klebsiella pneumonia* (*K. pneumonia* ATCC 13883), and *Pseudomonas aeruginosa* (*P. aeruginosa* ATCC 10145) using XTT examination. *Vancomycin,* as an example of a classical antibacterial medication, was employed for a comparison.

**Figure 9:**
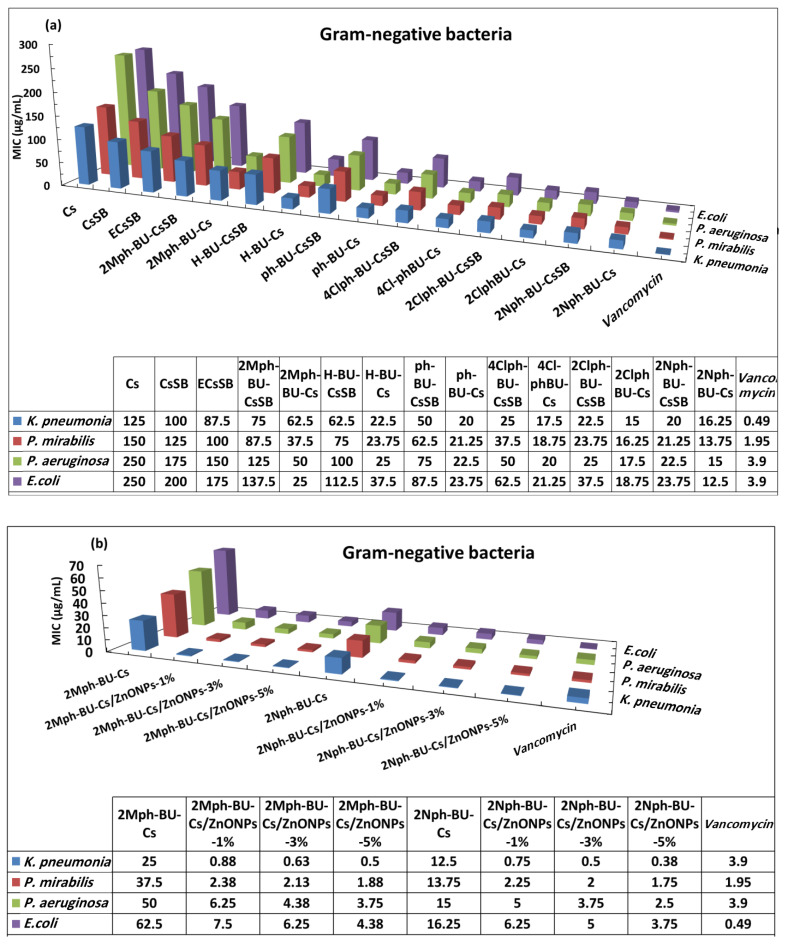
MIC index for: (**a**) R-BU-CsSB and R-BU-Cs hydrogels, and (**b**) 2Nph-BU-Cs/ZnONPs and 2Mph-BU-Cs/ZnONPs composites against the inspected Gram-negative bacteria.

**Figure 10:**
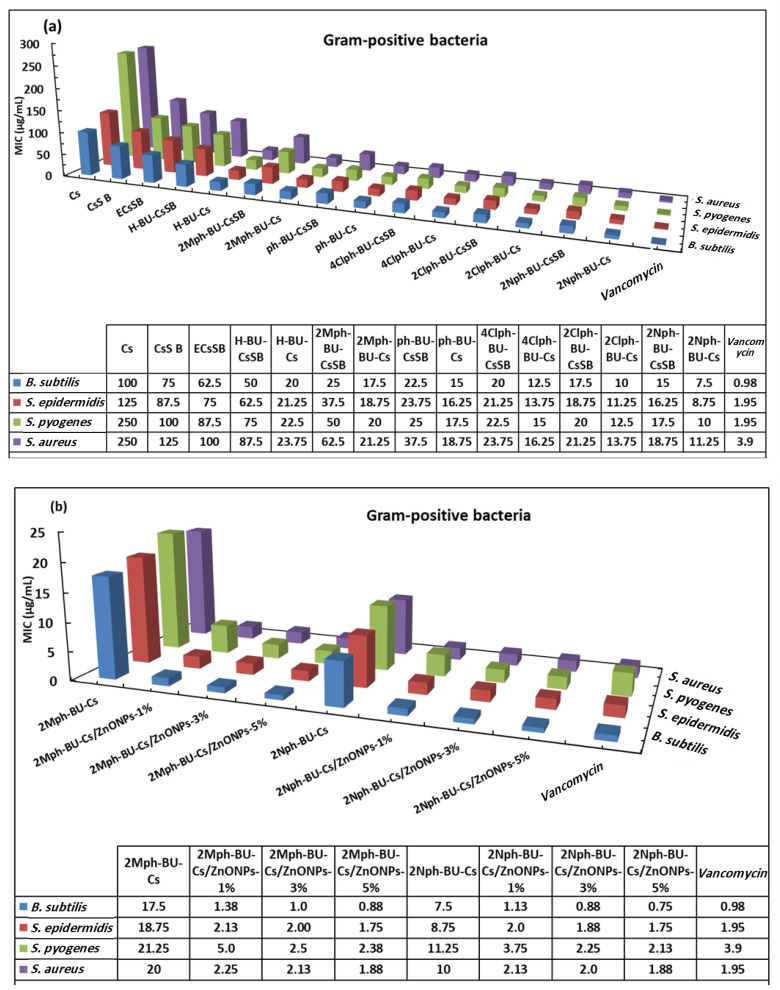
MIC index for: (**a**) R-BU-CsSB and R-BU-Cs hydrogels and (**b**) 2Nph-BU-Cs/ZnONPs and 2Mph-BU-Cs/ZnONPs composites against the inspected Gram-positive bacteria.

The findings demonstrated that the prepared R-BU-CsSB and R-BU-Cs hydrogels had much more antibacterial activity compared to virgin Cs (Figure 9a and Figure 10a).

There have already been proposed three action methods for explaining how chitosan inhibits bacterial growth. The most logical one is that polyanionic bacterial cell membranes and polycationic chitosan chains interact together electrostatically. Thus, the internal electrolytes and protein-rich components of the bacterial cells are lost as a result of changing the permeability of their membranes [35,42]. According to this hypothesis, the materials’ antibacterial effectiveness will be enhanced as the number of cationized sites on them grows. Compared to the chitosan’s structure, R-BU-CsSB hydrogels include -C=O, -N=CH, -NH, -NCH_3_, -CO-N-CH, and -OH as more sites, whereas R-BU-Cs hydrogels comprise -C=O, -NH, -NCH_3_, -CO-N-CH, and -OH in addition to the regained -NH_2_ groups. Due to the ease with which the aforementioned polar groups may be protonated, the R-BU-CsSB and R-BU-Cs hydrogels have a significantly higher number of cationic sites. As a consequence, the net result of their positive charge intensifies, their electrostatic contact with the membranes of bacterial cells that have negative charge sites grows, and as a result, the activity against bacteria is enhanced [32].

According to the second hypothesized mechanism, chitosan combines with microbe’s DNA and prevents the production of protein and mRNA [43]. The separation between the chains in R-BU-CsSB and R-BU-Cs hydrogels predominates. This is explained by the incorporation of both the benzaldehyde pendant moieties and R-BU linkages in R-BU-CsSB hydrogels and the inclusion of R-BU linkages in R-BU-Cs hydrogels. As a result, the attraction forces between chains greatly decreased, which aided their entry into the microorganisms’ cells, leading to a better antibacterial effect by preventing DNA from being turned into RNA, which inhibits the microbes from growing.

The third mechanism was hypothesized in reliance on the distinctive binding ability of chitosan with metal salts, significant nutritive ingredients, and spore elements [44]. Compounds with imine groups and R-BU linkages have long been recognized as acting as efficient ligands for chelating metals [32,45]. As a result, the insertion of these linkages into R-BU-CsSB and R-BU-Cs hydrogels improves their chelating sites for key nutrients, metal salts, and spore components. This explains why the investigated hydrogels are more effective than their parent Cs in killing germs.

R-BU-Cs hydrogels perform better in inhibiting all the tested bacteria compared to R-BU-CsSB hydrogels. The MIC values of R-BU-Cs hydrogels varied from 7.5 to 62.5 μg/mL, whereas the MIC values of R-BU-CsSB hydrogels ranged from 15 to 137.5 μg/mL. This is ascribed to the R-BU linkages beside the restored primary -NH_2_ groups which can be facilely converted to their protonated states. The latter can easily bind with anionized sites on the membranes of the bacterial cells by electrostatic interactions. This affirms the most agreeable assumption for the chitosan capacity as an antibacterial agent. R-BU-Cs hydrogels are characterized by their higher inhibition potency than R-BU-CsSB hydrogels, referring to the greater inhibitory capacity of the primary -NH_2_ groups against the bacterial activity relative to that of the imino groups. This is ascribed to the fact that the protonation of the primary -NH_2_ groups is proceeded easier and to a higher extent than that of the imine groups, enhancing their electrostatic interaction performance with the negative charged sites on the membranes of the bacterial cells and boosting their activities against bacteria relative to that of R-BU-CsSB hydrogels.

Moreover, the inhibition effectiveness of the R-BU-Cs and R-BU-CsSB hydrogels against the tested Gram-negative bacteria might be varied in the following ways: *K. pneumonia* > *P. mirabilis* > *P. aeruginosa* > *E. coli* (Figure 9a). While, their effectiveness in reducing Gram-positive bacteria activity may be summarized as follows: *B. subtilis* > *S. epidermidis* > *S. aureus* > *S. pyogenes* (Figure 10a).

Further, both R-BU-Cs and R-BU-CsSB hydrogels do better in inhibiting Gram-positive bacteria than Gram-negative ones. The MIC values of R-BU-Cs hydrogels varied from 7.5 to 23.75 μg/mL and from 12.5 to 62.5 μg/mL, whereas the MIC values of R-BU-CsSB hydrogels ranged from 15 to 87.5 μg/mL and from 20 to 137.5 μg/mL against the Gram-positive bacteria (Figure 10a) and Gram-negative ones, respectively (Figure 9a). The structure of the walls of the Gram-positive bacterial cells differs from the Gram-negative ones; whereas the first is distinguished by a porous nature which facilitates the penetration of the external substances within their cells, the Gram-negative bacteria is comprised of a complex two-layer walls (a thick outer layer and a thin inner layer). The exterior layer behaves as a barrier for obstructing the external substances to be penetrated into the cells [46]. Consequently, the variance in the structures of these two kinds of bacteria is accountable for the difference in the inhibition potency of the R-BU-Cs and R-BU-CsSB hydrogels.

The inhibitory action of the prepared hydrogels is considerably influenced by the type of the substituent group in the aryl moiety of the BU derivatives, as shown in Figure 9a and Figure 10a. The results indicate that BU derivatives can be assorted in accordance with their inhibition efficiencies into two major classes, between which lies the unsubstituted derivative. To the first class, distinguished by a higher inhibition potency comparative to that of the unsubstituted derivative, belongs the BU derivative of an electron-poor substituent (-NO_2_, -Cl) which reduces the electrons’ density on its derivative. On the other hand, the derivatives having lower inhibitory action comparative to that of the unsubstituted derivative possess an electron-rich substituent (-OMe) that can increase the electrons’ density on its derivative. Experimental evidence boosting these conclusions has been affirmed by the higher inhibition potency of the nitro- and chloro- hydrogels compared to that of the methoxy hydrogel. This is in accordance with the higher electron-withdrawing capacity of the nitro- and chloro- groups relative to methoxy group. Moreover, there is another evidence shown from the greater potency of the chloro- group in position two compared to its inhibitory effect when it is in position four in the aryl moiety of the BU. Because in the first case, electron withdrawing is much faster and consequently easier.

Although 2Nph-BU-Cs is the most effective hydrogel in inhibiting the activity of the tested bacteria in comparison to the other hydrogels, its level of inhibition is less effective than that of the medication *Vancomycin.* Thus, 2Nph-BU-Cs and 2Mph-BU-Cs, having the highest and the lowest inhibition performance against the tested bacterial activity, respectively, have been chosen to create some ZnONPs composites to reinforce their antibacterial action.

As would be predicted, the MIC values of the 2Nph-BU-Cs/ZnONPs-1%, 2Nph-BU-Cs/ZnONPs-3%, and 2Nph-BU-Cs/ZnONPs-5% against all the tested Gram-negative bacteria which ranged from 0.38 to 6.25 μg/mL (Figure 9b) and Gram-positive bacteria which ranged from 0.75 to 1.13 μg/mL (Figure 10b) were substantially lower than the MIC values of 2Nph-BU-Cs free from ZnONPs (ranged from 12.5 to 16.25 μg/mL and from 7.5 to 10 μg/mL, respectively), Similarly, MIC values of 2Mph-BU-Cs/ZnONPs-1%, 2Mph-BU-Cs/ZnONPs-3%, and 2Mph-BU-Cs/ZnONPs-5% ranged from 0.5 to 7.5 μg/mL and from 0.88 to 5 μg/mL against Gram-negative and Gram-positive bacteria, respectively, which were significantly lesser than that of their parent 2Mph-BU-Cs (ranging from 25 to 62.5 μg/mL and from 17.5 to 20 μg/mL, respectively), as shown in Figure 9b and Figure 10b, respectively.

Moreover, for both the 2Nph-BU-Cs/ZnONPs and 2Mph-BU-Cs/ZnONPs composites, the increment of the inserted ZnONPs from 1% to 5% resulted in improvement in the inhibitory efficacy of the ZnONPs bio-composites against all the kind of examined Gram-negative and Gram-positive bacteria as illustrated in Figure 9b and Figure 10b, respectively. In comparison to the typical medicine *Vancomycin*, both 2Nph-BU-Cs/ZnONPs and 2Mph-BU-Cs/ZnONPs composites have shown much stronger activity against most of the tested Gram-negative bacteria (Figure 9b) and all tested Gram-positive bacteria (Figure 10b).

It was established that ZnONPs can directly contact with the bacterial cell wall, release the antibacterial Zn^+2^ ions and the reactive oxygen species which destroy the bacterial walls [26,47], and increase of the permeability of the cell membrane, letting the penetration of the nanoparticles into the bacterial cytoplasm [47]. Therefore, the synthesized ZnONPs bio-composites might be a promising option to antibiotics, particularly against bacterial strains resistant to standard medications.

### 3.4. Antifungal Activity

By employing the micro-dilution technique via XTT examination, the antifungal activity of the Cs, CsSB, ECsSB, R-BU-CsSB, and R-BU-Cs hydrogels as well as the ZnONPs bio-composites was evaluated against some fungi, specifically *Aspergillus fumigatus* (*A. fumigatus* ATCC 9197), *Aspergillus niger* (*A. niger* ATCC 6275), *Cryptococcus neoformans* (*C. neoformans* ATCC 66031), and *Candida albicans* (*C. albicans* ATCC 18804). For comparison, *Amphotericin B*, a common antifungal medication was used.

**Figure 11:**
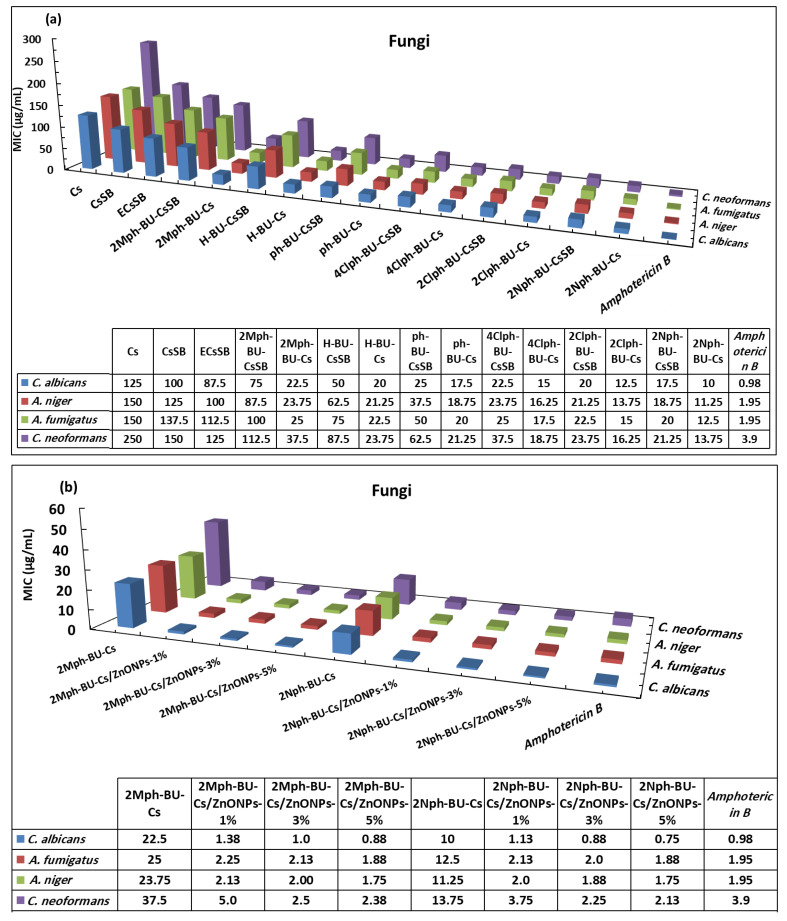
MIC index for: (**a**) R-BU-CsSB and R-BU-Cs hydrogels and (**b**) 2Nph-BU-Cs/ZnONPs and 2Mph-BU-Cs/ZnONPs composites against the inspected fungi.

All the prepared R-BU-CsSB and R-BU-Cs hydrogels exhibited superior inhibitory activity against the investigated fungi in comparison to the parent chitosan (Figure 11a). Similar to the effects shown in bacterial cells, the mechanism of chitosan’s antifungal action involves the formation of the cell wall’s structural properties while chitosan molecules directly inhibit fungal growth. The activity of the enzymes necessary for fungus growth is hampered by the diffusion of chitosan molecules into the hyphae, as demonstrated by microscopic investigations [48].

Again, all of the R-BU-Cs hydrogels showed a higher antifungal activity in comparison with their corresponding R-BU-CsSB hydrogels (Figure 11a).

Further, the inhibition performance of the R-BU-Cs and R-BU-CsSB hydrogels against the inspected fungi can be arranged as: *C. albicans* > *A. niger* > *A. fumigatus* > *C. neoformans* (Figure 11a). This is due to the difference between the structure of cell walls for various fungi strains.

Once more, the results indicated the better inhibition potency of the nitro- and chloro- hydrogels comparative to that of the methoxy hydrogel (Figure 11a).

As seen from the MIC values, the impregnation of ZnONPs into the 2Nph-BU-Cs and 2Mph-BU-Cs matrices significantly increased the effectiveness of the produced nano bio-composites toward suppressing the activity of all the tested fungal strains (Figure 11b). MIC values for 2Nph-BU-Cs/ZnONPs-1%, 2Nph-BU-Cs/ZnONPs-3%, and 2Nph-BU-Cs/ZnONPs-5% ranged from 3.75 to 0.75 µg/mL against all the fungi that were tested, which were lower than the MIC values of 13.75 to 10 µg/mL for their parent 2NphBU-Cs. In addition, 2Mph-BU-Cs/ZnONPs-1%, 2Mph-BU-Cs/ZnONPs-3%, and 2Mph-BU-Cs/ZnONPs-5% showed MIC values ranging from 5 to 0.88 µg/mL that were lower than those of their parent 2Mph-BU-Cs (ranged from 22.5 to 37.5 µg/mL).

When the amount of ZnONPs was increased from 1% to 5%, there was a noticeable improvement in the bio-composites’ ability to restrict the fungal growth. Further, 2Nph-BU-Cs/ZnONPs-5% and 2Mph-BU-Cs/ZnONPs-5% had more inhibition effectiveness against all of the investigated fungi than the common medication *Amphotericin B.* (Figure 11b). The boosted inhibitory action of the ZnONPs bio-composites can be ascribed to the synergistic interaction between the antifungal activity of ZnONPs and 2Nph-BU-Cs/or 2Mph-BU-Cs in their nano composites. Therefore, ZnONPs bio-composites are a potential antibiotic substitute, especially against the fungal strains that are resistant to conventional medications.

### 3.5. Cytotoxicity Evaluation

**Figure 12:**
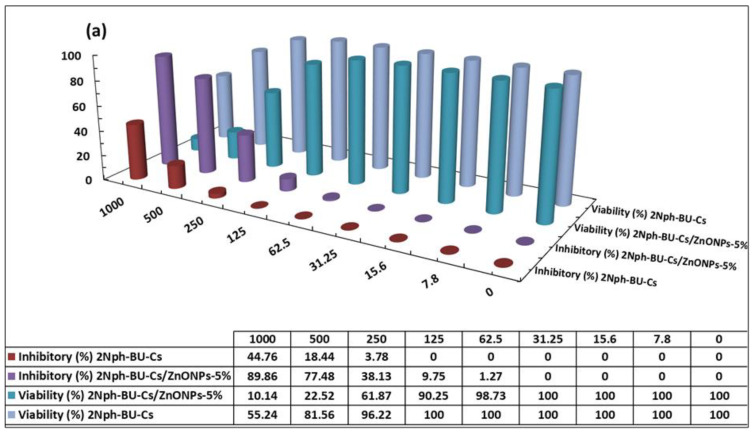

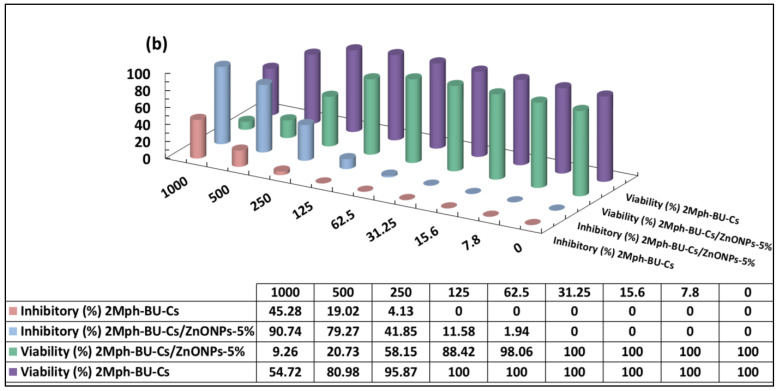
Cytotoxic impact of the prepared nano bio-composites on MRC-5 cells line: (**a**) 2Nph-BU-Cs/ZnONPs-5% and (**b**) 2Mph-BU-Cs/ZnONPs-5%.

MRC-5 cells, normal human lung fibroblast cells line, were used to explore the cytotoxic impact of 2Nph-BU-Cs, 2Mph-BU-Cs, and their ZnONPs-5% bio-composites using a concentration range of 1000–0 µg/mL, and the results are represented in Figure 12 (a and b, respectively). It could be noted that the cells’ viability was not influenced by both 2Nph-BU-Cs and 2Mph-BU-Cs at a concentration ≤ 125 µg/mL, while it was slightly affected at a concentration of 250 µg/mL, where the inhibition percent was less than 5% (3.78 and 4.13% for 2Nph-BU-Cs and 2Mph-BU-Cs, respectively) as shown in Figure 12 (a and b, respectively). Further, CC_50_, the half-calculated cytotoxic concentration that leads to a poisonous impact on 50% of intact cells, of both 2Nph-BU-Cs and 2Mph-BU-Cs was >1000 µg/mL. The MIC values of both the 2Nph-BU-Cs and 2Mph-BU-Cs hydrogels against the tested bacteria and fungi, illustrated in Figure 9a, Figure 10a and Figure 11a, were found to be much lower than their cytotoxic concentration (250 µg/mL). This indicates a safe usage of 2Nph-BU-Cs and 2Mph-BU-Cs on the normal cells of human, permitting them for applications in the biomedicine and pharmaceutical domains.

**Figure 13:**
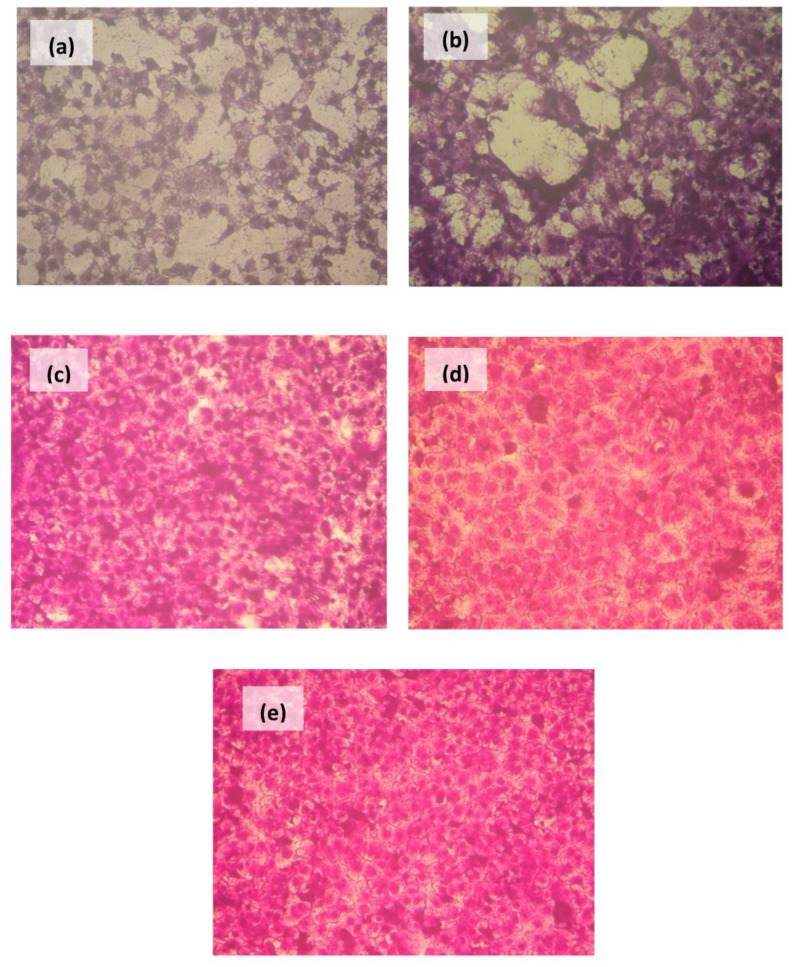
Microscopic examination of MRC-5 cells treated with 2Nph-BU-Cs/ZnONPs-5% at different concentrations: (**a**) 1000 µg/mL, (**b**) 500 µg/mL, (**c**) 250 µg/mL, (**d**) 125 µg/mL, compared with (**e**) non-treated MRC-5 cells (control). All the images were obtained at a magnification of 100×.

On the other hand, the viability of the MRC-5 cells wasn’t affected by both 2Nph-BU-Cs/ZnONPs-5% and 2Mph-BU-Cs/ZnONPs-5% composites at a concentration ≤31.25 µg/mL, while it was slightly affected at a concentration of 62.5 µg/mL, where the inhibition percent was less than 2% (1.27 and 1.94% for 2Nph-BU-Cs/ZnONPs-5% and 2Mph-BU-Cs/ZnONPs-5% composites, respectively) as shown in Figure 12 (a and b, respectively). Moreover, the CC_50_ of both the 2Nph-BU-Cs/ZnONPs-5% and 2Mph-BU-Cs/ZnONPs-5% composites was 300 ± 3.35 and 290 ± 4.15 µg/mL, respectively. Again, the MIC data of these nanocomposites against the examined bacteria and fungi (Figure 9b, Figure 10b and Figure 11b) show that they were much lower than their cytotoxic concentration (62.5 μg/mL). This suggests that these nanocomposites are secure on the normal cells of humans and can be used in the biomedical fields. The microscopic examination of the MRC-5 cells that have been treated with different concentrations of the 2Nph-BU-Cs/ZnONPs-5% composite and incubated for 24 h is shown in Figure 13.

## 4. Conclusions

From the obtained results, one can provide the following conclusions:

1. Various substituted bisuracil (R-BU) derivatives are efficient crosslinkers for binding chitosan Schiff’s base chains (R-BU-CsSB) and chitosan chains (R-BU-Cs).
2. A simple and easy multi-step method was utilized for chemical cross-linking processes; first, by a condensation of the bioactive 1^ry^ amino groups of chitosan with benzaldehyde molecules to yield CsSB and thus protecting them from reaction with epichlorohydrin; second, by a selective reaction between the 1^ry^ hydroxyl groups at C6 of the obtained CsSB and epichlorohydrin to produce ECsSB; third, by a facile opening of the epoxy rings of the latter via a reaction with the amino groups of R-BU derivatives to obtain crosslinked polymeric matrices containing additional bioactive nitrogen-rich BU moieties (R-BU-CsSB hydrogels); and fourth, by elimination of the benzaldehyde moieties of the latter to retrieve the bioactive amino groups on chitosan, producing R-BU-Cs hydrogels.
3. The more reactive and biologically active amino groups on chitosan did not consume because the modifications were confined on 1^ry^ -OH groups at C6 on chitosan.
4. Dispersion of three various amounts of ZnONPs (1, 3, and 5% based on the hydrogel weight) inside 2Nph-BU-Cs and 2Mph-BU-Cs hydrogels led to the formation of six ZnONPs bio-composites. Several proper analytical methods proved the successful formation of these composites.
5. The inhibition potency of R-BU-CsSB and R-BU-Cs hydrogels as well as the ZnONPs bio-composites against all the tested microbes were arranged as: ZnONPs bio-composites > R-BU-Cs hydrogels > R-BU-CsSB hydrogels > Cs.
6. Both R-BU-Cs and R-BU-CsSB hydrogels as well as the ZnONPs bio-composites do better in inhibiting Gram-positive bacteria than Gram-negative ones. MIC values were influenced by the type and position of substituents in the aryl ring of R-BU linkages in the hydrogels being lower for the substituents of negative resonance effect (−R) and higher for those of positive resonance effect (+R).
7. Compared with the used standard antibiotics, both 2Nph-BU-Cs/ZnONPs and 2Mph-BU-Cs/ZnONPs composites have shown much stronger inhibition activity against most of the inspected Gram-negative bacteria, all the tested Gram-positive ones, and all of the investigated fungi.
8. 2Nph-BU-Cs, 2Nph-BU-Cs/ZnONPs-5%, 2Mph-BU-Cs, and 2Mph-BU-Cs/ZnONPs-5% are not hazardous to normal human cells.
9. Incorporation of R-BU linkages and ZnONPs into chitosan strengthens its inhibitory action on the growth of hazardous microorganisms. This represents an appropriate technique for obtaining promising compounds that can effectively compete with common antibiotics.

Based on the above, it is possible to recommend the use of the prepared hydrogels and their ZnONPs as antimicrobial agents. For prospective expectation, we will study the possibility of their use as antioxidants, anti-inflammatories, antivirus, antitumor, and adsorbents for the removal of acidic pollutants from their effluents for industrial wastewater treatment.

## Figures and Tables

**Table 1:** Elemental analysis of the R-BU-CsSB and R-BU-Cs hydrogels.

Sample Code	Elemental Analysis				
	%C	%H	%N	%O	%Cl
Cs	44.90	6.86	8.61	39.63	-
CsSB	62.80	5.98	5.70	25.52	-
ECsSB	63.08	6.11	4.48	26.33	-
2Nph-BU-CsSB	58.30	5.63	11.89	24.18	-
2Nph-BU-Cs	50.56	5.79	14.47	29.18	-
2Clph-BU-CsSB	58.62	5.65	10.79	21.52	3.42
2Clph-BU-Cs	51.14	5.83	13.00	25.80	4.23
4Clph-BU-CsSB	58.55	5.68	10.78	21.54	3.45
4Clph-BU-Cs	51.09	5.80	12.98	25.97	4.16
Ph-BU-CsSB	60.80	5.94	11.09	22.17	-
Ph-BU-Cs	53.45	6.24	13.46	26.85	-
2Mph-BU-CsSB	59.39	6.38	10.90	23.33	-
2Mph-BU-Cs	53.11	6.19	12.92	27.78	-
H-BU-CsSB	57.79	6.08	12.10	24.03	-
H-BU-Cs	49.09	6.35	14.85	29.71	-

## Data Availability

The data presented in this study are available on request from the corresponding authors.
